# Intraarticular arthrofibrosis of the knee alters patellofemoral contact biomechanics

**DOI:** 10.1186/s40634-017-0110-8

**Published:** 2017-12-19

**Authors:** Jacob D. Mikula, Erik L. Slette, Kimi D. Dahl, Scott R. Montgomery, Grant J. Dornan, Luke O’Brien, Travis Lee Turnbull, Thomas R. Hackett

**Affiliations:** 1 Steadman Philippon Research Institute, 181 W. Meadow Drive, Suite 1000, Vail, CO 81657 USA; 2 The Steadman Clinic, 181 W Meadow Dr, Ste 400, Vail, CO 81657 USA; 3 Howard Head Sports Medicine, 180 S Frontage Rd W, Vail, CO 81657 USA

**Keywords:** Arthrofibrosis, Suprapatellar, Anterior interval, Patellofemoral contact force, Anterior knee pain

## Abstract

**Background:**

Arthrofibrosis in the suprapatellar pouch and anterior interval can develop after knee injury or surgery, resulting in anterior knee pain. These adhesions have not been biomechanically characterized.

**Methods:**

The biomechanical effects of adhesions in the suprapatellar pouch and anterior interval during simulated quadriceps muscle contraction from 0 to 90° of knee flexion were assessed. Adhesions of the suprapatellar pouch and anterior interval were hypothesized to alter the patellofemoral contact biomechanics and increase the patellofemoral contact force compared to no adhesions.

**Results:**

Across all flexion angles, suprapatellar adhesions increased the patellofemoral contact force compared to no adhesions by a mean of 80 N. Similarly, anterior interval adhesions increased the contact force by a mean of 36 N. Combined suprapatellar and anterior interval adhesions increased the mean patellofemoral contact force by 120 N. Suprapatellar adhesions resulted in a proximally translated patella from 0 to 60°, and anterior interval adhesions resulted in a distally translated patella at all flexion angles other than 15° (*p* < 0.05).

**Conclusions:**

The most important finding in this study was that patellofemoral contact forces were significantly increased by simulated adhesions in the suprapatellar pouch and anterior interval. Anterior knee pain and osteoarthritis may result from an increase in patellofemoral contact force due to patellar and quadriceps tendon adhesions. For these patients, arthroscopic lysis of adhesions may be beneficial.

## Background

**Fig. 1:**
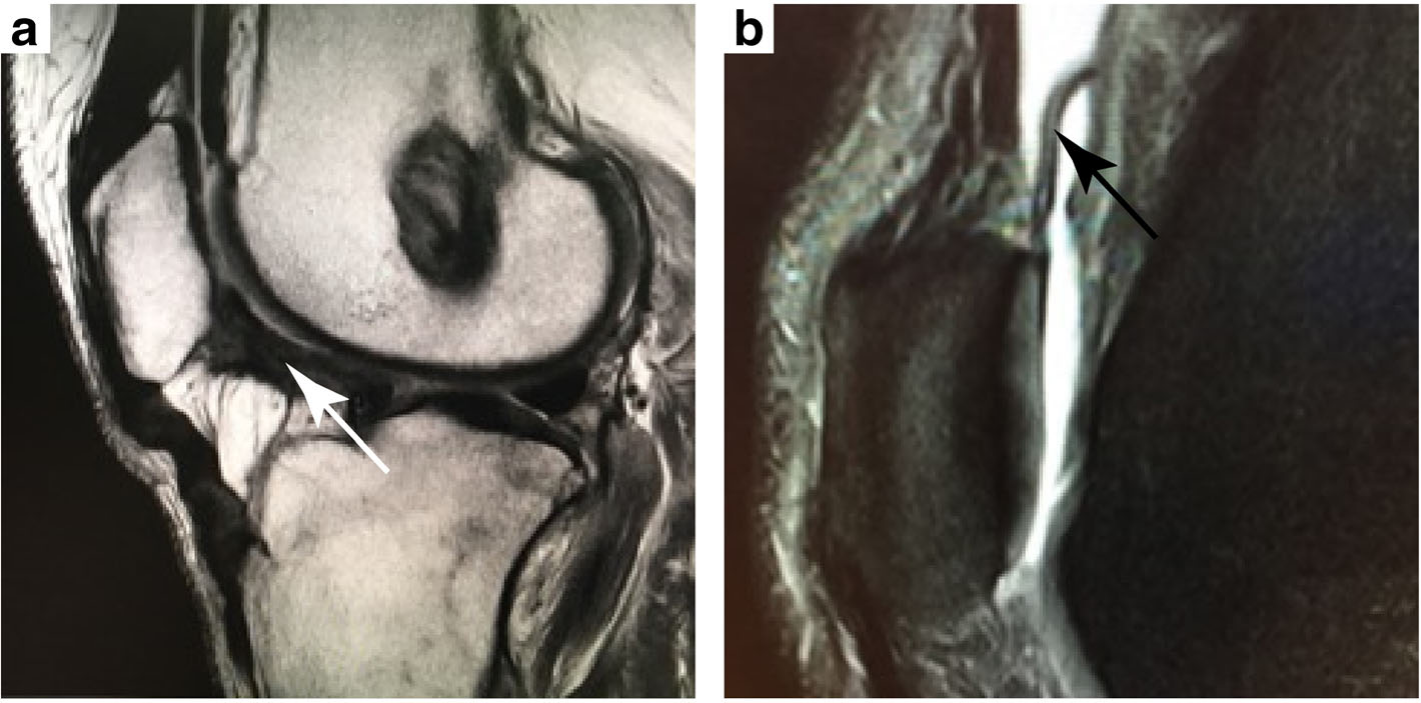
MRI images showing arthrofibrotic adhesions (arrows) of the **a** anterior interval and **b** suprapatellar pouch

Intraarticular arthrofibrosis (adhesions) remains a frequent complication following knee trauma or surgery (Petsche and Hutchinson 1999; DeHaven et al. 2003; Cosgarea et al. 1994). Arthrofibrosis is defined as the development of excessive fibrotic tissue within a joint that leads to contracture and reduction in knee flexion, extension, and patellar mobility (Shelbourne et al. 1996; Murakami et al. 1997). In the knee, arthrofibrosis can occur in the anterior interval, the infrapatellar fat pad, the pretibial recess, and the suprapatellar pouch (Fig. 1) (Enad 2014). The effects of adhesions in the anterior interval, defined as the space between the infrapatellar fat pad and anterior border of the tibia (Dragoo et al. 2010), have been investigated (Ahmad et al. 1998) but not fully biomechanically characterized. Additionally, there is a paucity of published literature on the effects of adhesions in the suprapatellar pouch, which is a large, proximal continuation of the synovial membrane of the knee. Its proximal border is not precisely defined in the literature, but has been estimated to be “2–3 finger-widths” superior to the proximal pole of the patella (Dragoo and Abnousi 2008). The main clinical manifestations of adhesions in the suprapatellar pouch are pain, stiffness, and flexion contractures (Jerosch and Aldawoudy 2007; Millett and Steadman 2001; Fitzsimmons et al. 2010; Diduch et al. 1997; Schiavone Panni et al. 2009).

Arthrofibrosis can lead to a loss in capsular compliance (Dragoo et al. 2010) and may alter tension on the synovium of the joint capsule, resulting in increased patellofemoral contact forces (Ahmad et al. 1998). This is thought to be one of the causes of knee pain resulting from arthrofibrosis (Murakami et al. 1997; Steadman et al. 2008). Ahmad et al. estimated an increase in patellofemoral contact force with adhesions in the anterior interval given a decrease in the patellar-quadriceps tendon angle, defined as the angle formed by the quadriceps and patellar tendons; (Ahmad et al. 1998) however, these calculations were neither directly assessed nor validated through biomechanical testing. Furthermore, no studies have investigated contact force in the context of suprapatellar adhesions and the exact location of suprapatellar pouch adhesions has not been studied.

The purpose of this study was to analyze the biomechanical effects of adhesions in the suprapatellar pouch and anterior interval and corresponding patellofemoral contact forces during simulated quadriceps muscle contraction from 0° to 90° of knee flexion. Arthrofibrotic adhesions were hypothesized to increase the patellofemoral contact force and contact pressure in comparison to the control condition (no adhesions).

## Methods

### Intraoperative measurements

During routine knee arthroscopy for intraarticular arthrofibrosis in 14 consecutive patients (mean age: 45 ± 17 years, range: 15–65 years; mean height: 175.3 ± 8.8 cm; mean weight: 74.0 ± 13.5 kg; eight male, six female; nine right knee, five left knee) intraoperative distance measurements were made from the proximal-most midsagittal point of the trochlea to two landmarks: 1) the proximal-most midsagittal point of the joint capsule and 2) the midsagittal point of the suprapatellar adhesions. These measurements were made intraoperatively, consistent with the standard of care, using a common surgical measurement device (ACUFEX, Smith & Nephew, Inc., Andover, MA) inserted through the anteromedial portal and were later referenced to aid in-vitro placement of simulated suprapatellar adhesions for the biomechanical model. Diagnosis of arthrofibrosis was based initially upon the patient’s history and then confirmed by careful examination of the knee in regard to a reduction of range of motion in comparison to the contralateral side. Particular attention was paid to patellofemoral mobility and excursion. Often the diagnosis was confirmed by MRI by visualization of the presence of heterotopic fibrous bands within the joint (Fig. 1).

### Cadaveric specimen preparation

**Fig. 2:**
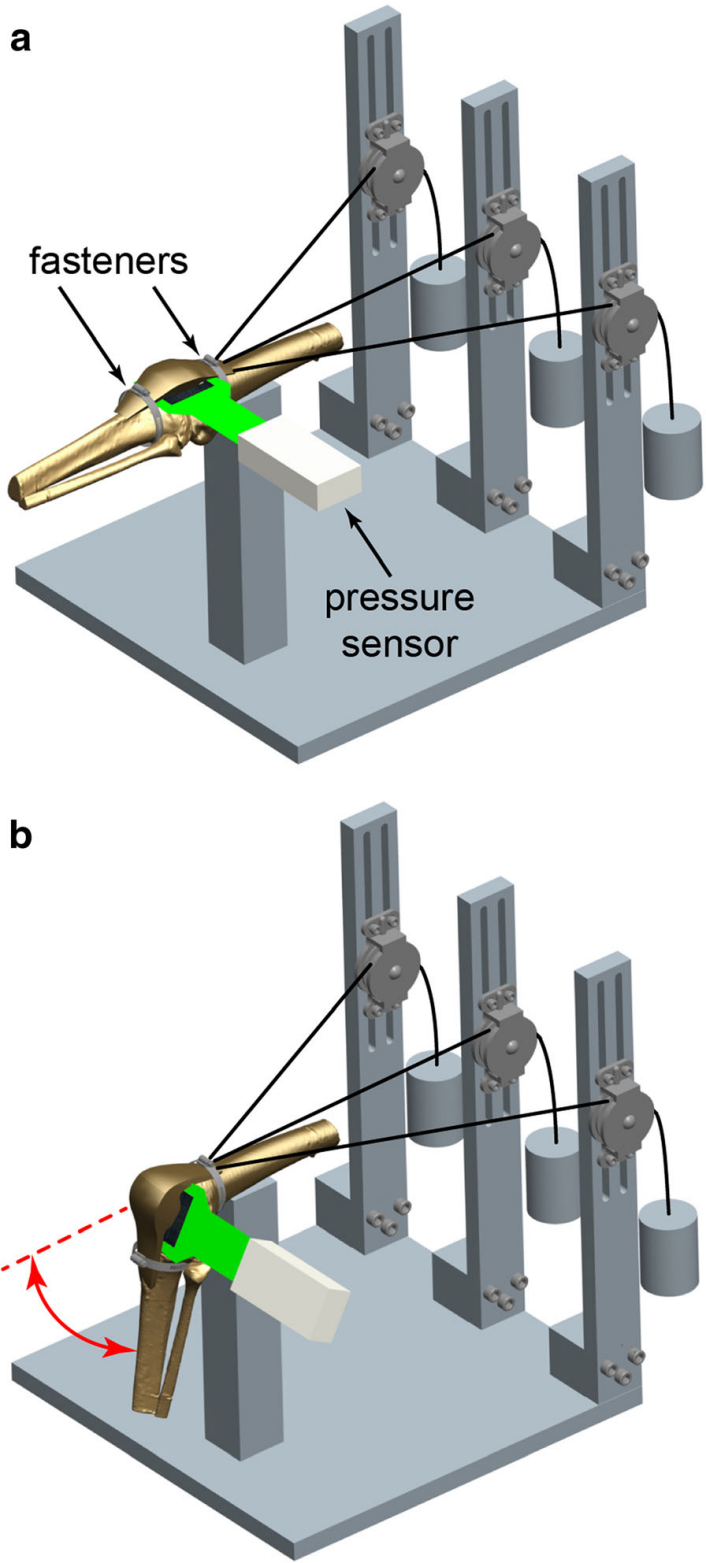
Schematics of the testing setup. Patellofemoral contact force was measured with a thin-film pressure sensor in response to simulated anterior interval, suprapatellar pouch, and combined anterior interval and suprapatellar pouch adhesions (fasteners) at **a** 0° and **b** through a range of knee flexion angles spanning 0°–90°

Ten fresh-frozen human cadaveric knees without prior injury, osteoarthritis, or surgical history (mean age: 50 years, range: 26–59 years; eight male, two female) were dissected free of skin and muscles while preserving tendons, ligaments, and capsular structures. The quadriceps tendon (medial, lateral, and proximal aspects) was sutured with #5 polyethylene/polyester suture (FiberWire, Arthrex Inc., Naples, FL) utilizing the modified triple Kessler technique, which has been shown to exhibit excellent strength during in-vitro biomechanical testing (Clanton et al. 2015). The proximal femur was rigidly secured in a custom fixture, and the distal tibia was rigidly fixed at specific flexion angles by a ring stand. Each group of quadriceps sutures was connected to weights through a system of pulleys to mimic the quadriceps muscle force (Fig. 2a). Individual muscle contributions were simulated as described in previous studies and included the vastus lateralis (178 N), vastus medialis obliquus (VMO; 89 N), and a combination of the vastus intermedius and rectus femoris (267 N) (Ahmad et al. 1998; Grood et al. 1984). Utilizing the long axis of the femur as the reference line and a goniometer for measurements, the suture representing the vastus lateralis was directed 15 degrees lateral, the suture representing the VMO was directed 50 degrees medial, and the suture representing the vastus intermedius and rectus femoris was directed 10 degrees medial (Lieb and Perry 1968). A small medial arthrotomy was performed on the knee immediately posterior to the patella, and a thin-film pressure sensor (Model 5051; Tekscan, Inc., South Boston, MA) was inserted between the patella and femur. Each pressure sensor was calibrated using associated software (I-Scan 6.10, Tekscan, Inc., South Boston, MA) and a two-point calibration method with low and high calibration point forces of 20 and 600 N, respectively, with a software sensitivity setting of 5, to encompass the expected forces and corresponding pressure readings based on pilot testing.

### Biomechanical testing

Suprapatellar and anterior interval adhesions were simulated by consistently tightening adjustable worm-drive fasteners (1.25-cm band-width) around the femur and tibia at the location of the respective suprapatellar pouch and anterior interval at each tested flexion angle. The fasteners were tightened to 1 in-lb. torque, such that the soft tissues were not over-constrained. As an experimental quality control step, a second “no adhesion” state was tested subsequent to the three adhesion states and compared with the first “no adhesion” state to confirm the fastener did not deform the tissue. Although fasteners do not perfectly re-create arthrofibrosis, extensive pilot testing confirmed adjustable worm-drive fasteners were the most reproducible biomechanical method for replicating intraarticular arthrofibrosis, especially when compared with other, less reproducible methods such as suture anchors. Furthermore, our experimental setup is analogous to a previously-validated knee arthrofibrosis model performed by Ahmad et al. 1998.

The center of the suprapatellar fastener was secured around the quadriceps tendon at the average measured location of the adhesions in the suprapatellar pouch (40 mm superior to the proximal aspect of the trochlea, as determined from the previously described intraoperative measurements). The center of the anterior interval fastener was secured around the patellar tendon midway between its origin on the inferior pole of the patella and its insertion on the tibial tuberosity. Knee flexion angles were randomized and, within each individual knee flexion angle, adhesion states were randomized to decrease any incremental testing bias while preventing pressure sensor migration resulting from flexion angle adjustments. Patellofemoral contact force, peak contact pressure, mean contact pressure, and patellar translation were measured at 0, 15, 30, 45, 60, 75, and 90° of knee flexion (Fig. 2b) under simulated quadriceps muscle activation via the previously described system of weights and pulleys. Patellar translation was calculated via a custom script (Matlab, The MathWorks, Inc., Natick, MA) as superior-inferior shifts in the centroid location of the recorded pressure map for suprapatellar and anterior interval adhesions states, relative to the no adhesions state, for each flexion angle.

### Statistical analysis

Patellofemoral contact force, mean contact pressure, and peak contact pressure were analyzed at each testing angle during simulated suprapatellar, anterior interval, and combined suprapatellar/anterior interval adhesions. The two “no adhesion” states mentioned previously as quality control checks indeed produced nearly identical results (on average within 0.01 N/mm^2^ average contact force and 0.07 N/mm^2^ of peak contact pressure; each *p*-value approximately 1) and thus all subsequent reporting only included the first “no adhesion” state. Furthermore, a global minimum threshold was applied to exclude all measured pressures less than 0.12 N/mm^2^ (corresponding to 0.2 N/sensel) in order to isolate patellar-trochlear contact with the thin-film pressure sensor. Visual inspection, in addition to a post-hoc sensitivity analysis of contact area and contact force, confirmed this minimum threshold successfully excluded soft tissue contact with the thin-film pressure sensor. As a simplification of the full linear mixed-effects model with repeated measures analysis, power calculations were conducted assuming dependent comparison of means (t-test), two-tailed testing and an alpha of 0.05. Ten specimens per group are sufficient to detect an effect size of d = 1.0 with 80% statistical power. This corresponds to the case where the paired mean difference is identical to the standard deviation of the differences.

Flexible two-factor linear mixed effects models were constructed to assess the effects of flexion angle and adhesion state on patellofemoral contact force, mean contact pressure, and peak contact pressure. Random intercepts were allowed for each specimen to account for the repeated measures nature of the study design. Models of varying complexity, allowing nonlinear relationships for flexion angle and interaction between the factors, were constructed and the optimal model was determined based on the Bayesian Information Criterion (BIC). Ultimately, a cubic model was found to best fit the relationship between flexion angle and patellofemoral contact force. Residual diagnostics were performed to confirm model assumptions and model fit. To address the primary hypotheses of the study, Tukey comparisons were made between adhesion states, and effect estimates were reported with 95% simultaneous confidence intervals. Lastly, the one-sample Wilcoxon signed-rank test was used to assess whether medial-lateral and proximal-distal translation significantly differed from the control (no adhesions) state at each flexion angle. The statistical software R, with packages *lme4*, *effects* and *multcomp*, was used for all analyses (R Core Team 2016; Hothorn et al. 2008).

## Results

### Intraoperative measurements

The mean distance from the trochlea to the proximal-most midsagittal point of the joint capsule was 68 ± 7 mm. The mean location of adhesions in the pouch was 40 ± 9 mm, which was approximately 60% of the distance from the trochlea to the end of the joint capsule.

### Simulated suprapatellar adhesions: patellofemoral contact biomechanics

**Fig. 3:**
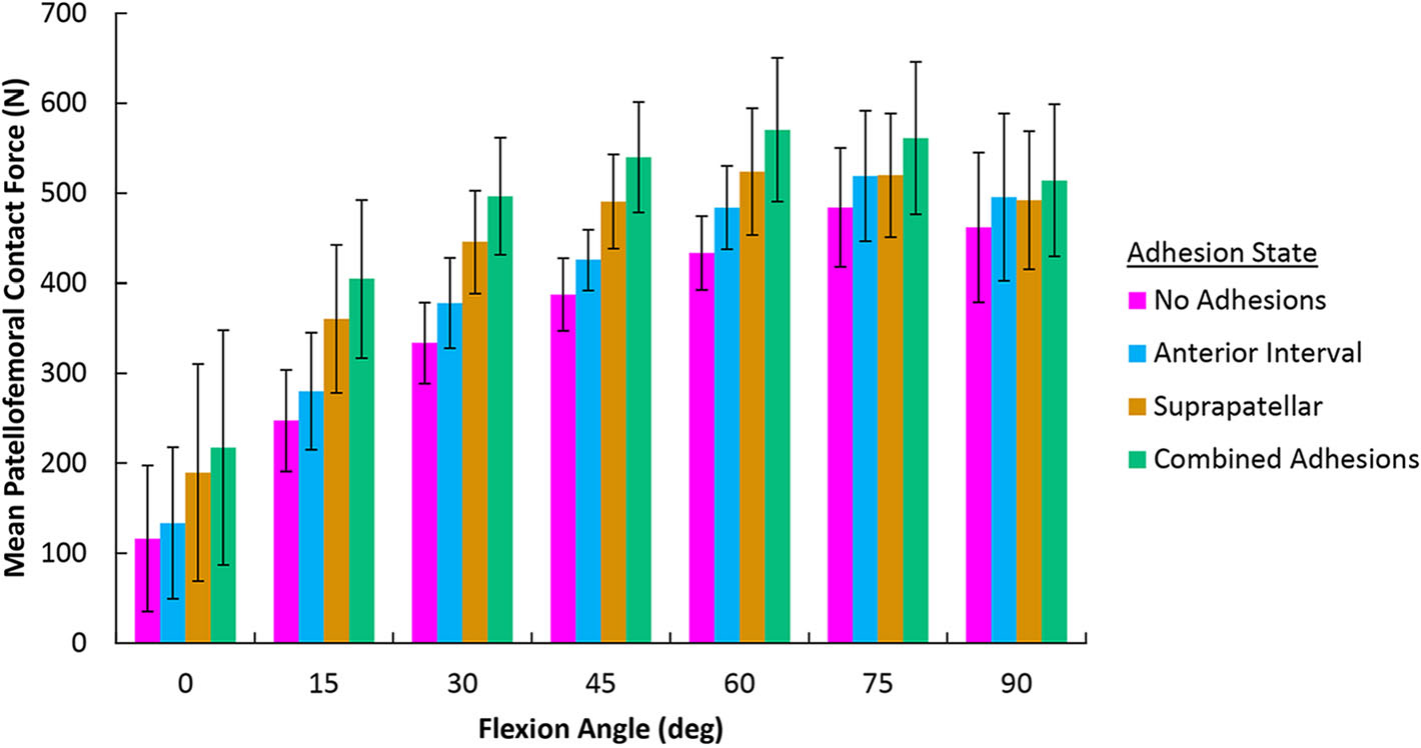
Patellofemoral contact force at each adhesion state (and across all flexion angles) was significantly different from the other adhesion states (*P* ≤ 0.007). Combined anterior interval adhesions resulted in the highest contact force followed by isolated suprapatellar adhesions, isolated anterior interval adhesions, and the no adhesions state

**Fig. 4:**
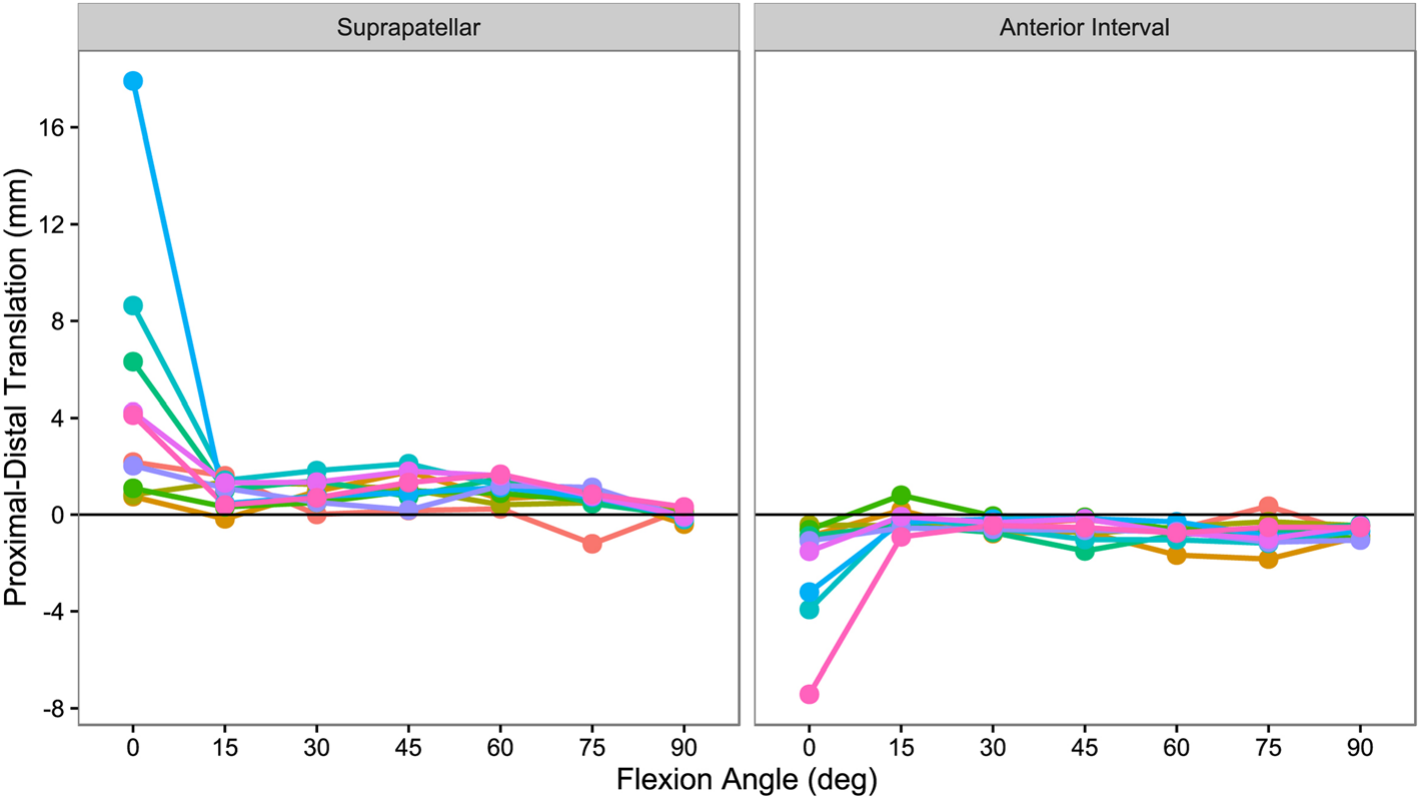
Proximal (+) and distal (−) translation of the patella with simulated adhesions. Suprapatellar adhesions tended to result in proximal patellar translation and anterior interval adhesions tended to result in distal translation, and these effects were most pronounced at 0° of knee flexion. Colored lines connect measurements made on the same specimen

Suprapatellar adhesions significantly increased the patellofemoral contact force across all flexion angles when compared to no adhesions by a mean of 80 N (95% CI [51, 109], *P* < 0.001). When compared to anterior interval adhesions, suprapatellar adhesions significantly increased contact force by a mean of 44 N (95% CI [15, 73], *P* < 0.001) (Fig. 3). In addition, suprapatellar adhesions resulted in a non-significant increase in the peak contact pressure between the patella and trochlea by a mean of 0.335 N/mm^2^ (95% CI [−0.048, 0.719], *P* > 0.119) and the mean contact pressure by a mean of 0.114 N/mm^2^ (95% CI [−0.004, 0.231], *P* > 0.063) across all flexion angles when compared to no adhesions. Suprapatellar adhesions caused significant proximal patellar translation at flexion angles from 0 to 60° (*P* < 0.05, each) (Fig. 4) and significant lateral translation at 0° (*P* = 0.014) when compared with the control group (no adhesions). The median proximal translation at 0° was 3.2 mm (range 0.8–17.9 mm) and the median lateral translation at 0° was 2.3 mm (range 1.0–3.8 mm).

### Simulated anterior interval adhesions: patellofemoral contact biomechanics

Anterior interval adhesions significantly increased the patellofemoral contact force across all flexion angles by a mean of 36 N (95% CI [7, 65], *P* = 0.007) when compared to no adhesions. In addition, anterior interval adhesions resulted in a non-significant increase in the peak contact pressure by a mean of 0.163 N/mm^2^ (95% CI [−0.221, 0.546], *P* > 0.776) and the mean contact pressure by a mean of 0.058 N/mm^2^ (95% CI [−0.059, 0.175], *P* > 0.667) between the patella and trochlea across all flexion angles when compared to no adhesions. Anterior interval adhesions caused significant distal patellar translation at all tested flexion angles except 15° (each *P* < 0.05) (Fig. 4). The median distal translation at 0° was 1.1 mm (range 0.4–7.4 mm). Anterior interval adhesions did not cause significant lateral or medial translation at any flexion angle.

### Simulated suprapatellar and anterior interval adhesions: patellofemoral contact biomechanics

Combined suprapatellar and anterior interval adhesions increased the patellofemoral contact force by 120 N (95% CI [91, 149], *P* < 0.001) across all flexion angles when compared to no adhesions. In addition, combined suprapatellar and anterior interval adhesions significantly increased patellofemoral contact force when compared to suprapatellar adhesions alone (mean = 40 N, 95% CI [11, 69], *P* = 0.001) and anterior interval adhesions alone (mean = 84 N, 95% CI [55, 113], *P* < 0.001). Combined suprapatellar and anterior interval adhesions significantly increased the peak contact pressure by a mean of 0.503 N/mm^2^ (95% CI [0.119, 0.886], *P* = 0.003) and mean contact pressure by a mean of 0.175 N/mm^2^ (95% CI [0.057, 0.292], *P* < 0.001) when compared to no adhesions.

## Discussion

The most important finding in this study was that adhesions in the suprapatellar pouch and anterior interval significantly increased patellofemoral contact forces, which affirmed our hypothesis. Adhesions in the suprapatellar pouch led to a larger increase in patellofemoral contact force when compared with anterior interval adhesions. When adhesions were created in both the anterior interval and suprapatellar pouch, the patellofemoral contact force was significantly greater than when isolated to either of the individual compartments. In vivo, this increase in contact force may lead to anterior knee pain in patients with arthrofibrosis. Therefore, arthroscopic lysis of adhesions in the suprapatellar pouch and/or anterior interval of the knee may reduce patellofemoral contact forces and concomitant anterior knee pain in patients with arthrofibrosis.

Despite the abundance of literature regarding arthrofibrosis of the knee, few studies have quantified the effects that arthrofibrosis may have on patellofemoral contact forces. Specifically, there is no biomechanical data on the effect of arthrofibrosis in the suprapatellar pouch. This study demonstrated that suprapatellar adhesions have a significant effect on patellofemoral contact forces. Furthermore, the increase in patellofemoral contact force with increasing flexion angle of the knee is consistent with previous studies (Rood et al. 2015; Beck et al. 2007; Nomura et al. 2005; Ostermeier et al. 2007). To date, few studies have been published on the outcomes of lysis of the adhesions in the suprapatellar pouch, but improvement in patient knee motion has been documented (Millett et al. 2001). Results from this study provide insight into the anatomy of the suprapatellar pouch and suggest that lysis of suprapatellar adhesions may decrease patellofemoral contact forces.

Similar to biomechanical findings in the suprapatellar pouch, arthrofibrosis in the anterior interval altered patellofemoral contact forces, which may result in chondral damage to the patella and trochlea (Steadman et al. 2008). Steadman et al. 2008 observed degeneration of the patellofemoral articular cartilage in all patients undergoing arthroscopic anterior interval release, in which the mean Lysholm score improved from 59 preoperatively to 81 postoperatively, and average International Knee Documentation Committee (IKDC) score improved from 49 to 70. After reviewing the literature, only one other study has assessed the biomechanical effect of anterior interval adhesions: Ahmad et al. 1998 estimated an increase in patellofemoral contact force with adhesions in the infrapatellar pouch given a posterior translation of the patellar tendon. However, in contrast to the current study, patellofemoral contact forces were not directly assessed during testing. The current study affirms Ahmad et al.’s prediction that adhesions in the anterior interval increase the patellofemoral contact force (Ahmad et al. 1998).

Abnormal positioning of the patella is another pathology reported in patients with intraarticular adhesions (Noyes and Barber-Westin 2017). The results of the current study suggest that adhesions in the suprapatellar pouch may lead to proximal translation of the patella, most prominently at 0 degrees of flexion. A proximal position of the patella relative to the trochlear groove is known as patella alta (Insall and Salvati 1971). This condition predisposes the affected knee to patellofemoral dislocation due to decreased contact with the femur, especially at lower flexion angles (Fox et al. 2012). The current study also observed distal translation of the patella with anterior interval adhesions, which aligns well with the results of Ahmad et al.’s study (Ahmad et al. 1998). Distal positioning of the patella in the trochlear groove is known as patella baja and is associated with anterior knee pain, extensor lag, and a reduction in range of motion (Fox et al. 2012; Flören et al. 2007).

This study was not without limitations. First, durability and sensitivity to temperature and fluids have been reported as potential sources of error when utilizing thin-film pressure sensors (Wilharm et al. 2013; Jansson et al. 2013). However, Wilharm et al. 2013 recently showed that retropatellar pressure can be reliably and repeatedly measured using thin-film sensors. Additionally, retropatellar placement of the pressure sensors necessitated a small arthrotomy on the knee. The current study utilized a medial arthrotomy because Rood et al. 2015 found that pressure sensor placement through medial arthrotomies did not have a significant effect on patellofemoral kinematics. The sample size for our intraoperative measurements was relatively small, but we felt it was sufficient to aid the placement of the suprapatellar fastener in our experiment. Finally, the experimental design and associated fastener which simulated adhesions was a simplified representation of in vivo arthrofibrosis. Nevertheless, the changes in patellofemoral contact forces observed in this study proved to be repeatable and, therefore, offer new insight related to the effect of suprapatellar and anterior interval adhesions on patellofemoral contact biomechanics.

## Conclusion

The most important finding in this study was that patellofemoral contact forces were significantly increased by adhesions in the suprapatellar pouch and anterior interval. This may lead to anterior knee pain in patients with advanced arthrofibrosis. Therefore, arthroscopic lysis of adhesions in the suprapatellar pouch and/or anterior interval of the knee may reduce patellofemoral contact forces and concomitant anterior knee pain in patients with arthrofibrosis.

## Abbreviations

VMO: Vastus medialis obliquus
